# Mycotic Abdominal Aortic and Iliac Aneurysm Caused by *Streptococcus equi* Subspecies *zooepidemicus* Bacteremia

**DOI:** 10.1055/s-0039-3401995

**Published:** 2020-02-19

**Authors:** Anass Madani, Clark J. Zeebregts, Alexander Lamprou, Ignace F. J. Tielliu

**Affiliations:** 1 Division of Vascular Surgery, Department of Surgery, University Medical Center Groningen, University of Groningen, Groningen, The Netherlands

**Keywords:** mycotic aneurysm, infectious aortitis, *Streptococcus equi*

## Abstract

This report describes a case of a taxidermist who presented with sepsis and excruciating back pain a few weeks after contact with a deceased horse. *Streptococcus equi* subspecies *zooepidemicus* (SESZ) was isolated from patient’s blood and two isolated mycotic aneurysms were found. The first was located in the distal abdominal aorta and the second in the right common iliac artery. Treatment consisted of penicillin administration for 6 weeks and surgical debridement of the infected tissue combined with autologous vein reconstruction. The patient was infection-free without complaints 1 year after discharge and the venous reconstruction was patent. Reports in literature of bacteremia with SESZ leading to the development of mycotic aneurysms are very scarce and show that prognosis is generally unfavorable.

## Introduction

*Streptococcus equi* (SE) is a group-C β-hemolytic zoonotic pathogen that rarely causes human infections. 1 Three subspecies of SE are recognized, including SE subspecies *zooepidemicus* (SESZ), SE subspecies *ruminatorum* (SESR), and SE subspecies *equi* (SESE). 2 Among the three subspecies, SESZ is the most common pathogen isolated in humans. Although this pathogen remains a rare cause of human infections, it has been associated with severe conditions, such as toxic shock syndrome, meningitis, arthritis, endocarditis, necrotizing myositis, aortitis, and mycotic aneurysms. 1 2 In domesticated animals, SESZ can colonize the airway resulting in either asymptomatic carriers or animals with upper respiratory infections, mastitis, or meningitis. 2 The most common mode of zoonotic transmission is attributed to animal contact, mostly to equine and cattle, and to the consumption of unpasteurized dairy products. 1 The other two subspecies of SE (SESR and SESE) are hardly ever isolated in human infections. 1

A mycotic aneurysm is a potentially life-threatening condition. Preexisting aneurysms can become infected or new aneurysms can develop as a result of infectious arteritis. 3 Septic embolisms originating from endocarditis are a notorious route of arteritis. 3 Recent evidence suggests the likelihood of endocarditis being the etiology of approximately 17 to 29% of all mycotic aneurysms. 3 4 The source of the infection could also be the result of direct dissemination from adjacent infected structures, such as the esophagus, duodenum, pericardium, or from an antecedent infection (such as pneumonia, urinary tract infection, cholecystitis, diverticulitis, or soft tissue infection). 4

Blood cultures are positive in 50 to 85% of mycotic aneurysms. 4 The most common pathogens are staphylococcus and salmonella species, with staphylococcus aureus being associated in 28 to 71% of the cases and salmonella in 15 to 24%. 3 This predominance of species is attributed to their high affinity for the arterial wall and especially to the diseased one. 3 Syphilis, tuberculosis, *Mycobacterium* , and *Streptococcus pneumoniae* are less common causes of mycotic aneurysms. 3 4 Lastly, in immune compromised patients or those on long-term broad-spectrum antibiotics, fungi can be responsible for mycotic aneurysms. 4

## Case Presentation

A 70-year-old male known with hypothyroidism and hypercholesterolemia presented to his general practitioner (GP) with a painful wound on the pulp of the right middle finger with suppuration for several days. On physical examination, an erythematous finger was seen with pus secretion and blisters. The patient was not aware of how he had acquired the wound. As a professional taxidermist, he regularly came into contact with a wide range of deceased animals ranging from wild swans to horses. His most recent project prior to the incident was preparing a horse. The GP started a short antibiotic regimen and the infection settled quickly.

Three weeks later, he suddenly developed fever combined with sharp back pain. The patient was admitted to a local hospital. The initial diagnosis was pyelonephritis. The urine culture, however, turned out to be negative, while the blood cultures were positive for group C streptococcus. The patient was treated with intravenous amoxicillin/clavulanic acid for 5 days and was discharged in a stable clinical condition.

The back pain, however, did not subside after discharge and gradually increased. The patient became septic and was readmitted with high suspicion of endocarditis.

**Fig. 1:**
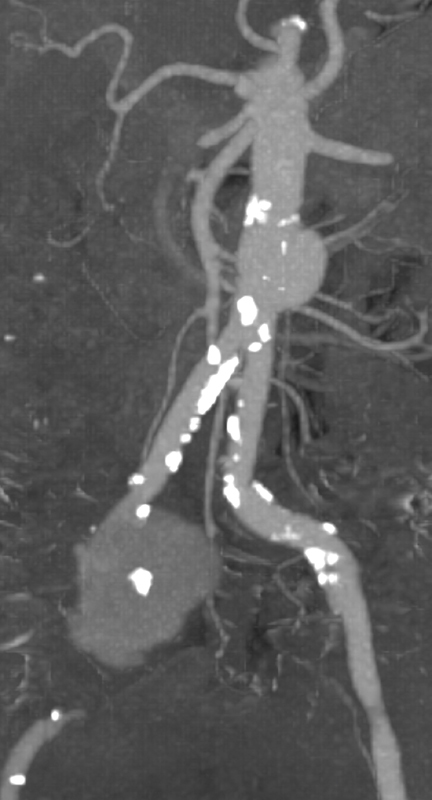
Maximum intensity projection of CTA showing both the isolated abdominal aortic and right common iliac artery aneurysm. CTA, computed tomography angiography.

During the workup, endocarditis was ruled out using transesophageal echocardiography. Duplex ultrasound examination (DUS) of the abdomen revealed an aneurysm of the abdominal aorta with wall-mounted thrombus formation. Further, magnetic resonance imaging (MRI) of the spine illustrated another isolated aneurysm in the right common iliac artery. Computed tomography angiography (CTA) confirmed the presence of the aneurysms in the distal abdominal aorta and in the right common iliac artery ( Fig. 1 ).

SESZ was isolated from the blood. Urine and fecal cultures were negative for bacterial growth.

The patient was then transferred to our tertiary referral center for further treatment. Upon arrival, a septic patient with excruciating back pain was seen. Blood analysis showed a white blood cell (WBC) count of 11.9 10E9/L (4–10 10E9/L), hemoglobin was 5.5 mmol/L (8.5–11 mmol/L), platelet count was 223 10E9/L (150–400 10E9/L), and C-reactive protein (CRP) was 77 mg/L (<5 mg/L).

The treatment plan consisted of a combined medical and surgical approach. Penicillin was given for a total duration of 6 weeks.

**Fig. 2:**
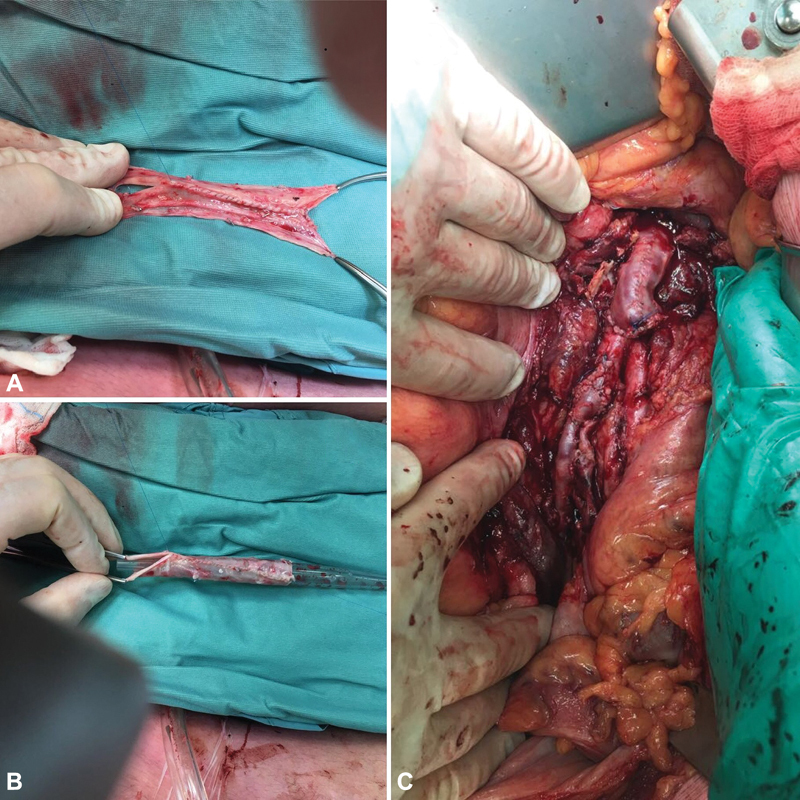
Back table construction of venous conduit for aortic reconstruction. Side to side anastomosis of two halves of the superficial femoral vein ( **A** ). Construction of tubular graft over a suction drain as a template ( **B** ). Intraoperative image of completed aortic and right iliac interposition graft with autologous veins ( **C** ).

Surgically, the infected tissue was debrided, followed by direct autologous vein reconstruction of the abdominal aorta and iliac artery using both superficial femoral veins. For this purpose the superficial femoral veins from both legs were harvested. For the aortic reconstruction, one vein was cut in half and the two halves were anastomosed to each other to form a tube with a larger diameter ( Fig. 2 ). For the iliac reconstruction, the other femoral vein was used. Both venous grafts were anastomosed in an end-to-end reconstruction.

The patient was readmitted to the hospital shortly after discharge with fever, shivering, malaise, and increased infection parameters, regardless of being treated with oral antibiotics. CTA demonstrated collections of fluid adjacent to the venous reconstruction. Serous fluid was percutaneously drained and cultured. The cultures were negative and the clinical condition dramatically improved directly after drainage. The patient was discharged and penicillin was continued orally at home.

The patient came back for follow-up 3 weeks after discharge and a new CTA showed a dramatic reduction of the fluid collections. Infection parameters, as follows, had decreased as well: WBC count of 4.5 10E9/L (4–10 10E9/L) and a CRP of 1.6 mg/L (<5 mg/L). During the last follow-up, 1 year after discharge, DUS of the abdomen showed an open venous reconstruction with no fluid collections and a CRP of 0.5 mg/L (<5 mg/L).

## Discussion

This report describes a rare incident of SESZ bacteremia and consequent development of mycotic aneurysms in the abdominal aorta and right common iliac artery, following contact with a deceased horse. The outcome was favorable for the patient after immediate surgical intervention and prolonged antibiotic administration.

Bacteremia with this pathogen has been associated with intravascular complications, such as endocarditis and aortitis. 2 There are, however, only a few cases reported of this pathogen being associated with the development of a mycotic aneurysms. 5

Yuen et al 6 reported 11 patients with SESZ septicemia in Hong Kong, of which only two presented with aortic mycotic aneurysms. The first patient did not survive despite receiving a bifurcated dacron graft along with intravenous amoxicillin and gentamicin administration. The second patient survived after receiving a bifurcated dacron graft alongside intravenous benzylpenicillin. The duration of antibiotic administration and follow-up were not reported for these two cases.

Altreuther et al 7 described two patients with SESZ bacteremia. The first presented with an infected bifurcated graft and the second presented with a symptomatic mycotic abdominal aortic aneurysm. The first patient responded well to prolonged antibiotic administration. The second patient was treated with endovascular aneurysm repair and prolonged antibiotics. Both patients were in a good clinical condition during follow-up at 4.5 years after discharge.

Lastly, Edwards et al 8 reported 11 cases of milk-borne SESZ bacteremia in England. Only one patient developed a mycotic aneurysm that was found in a femoral artery vein graft. This patient recovered fully after surgical replacement of the infected aneurysm with a graft and antibiotic administration.

SESZ is a group-C β-hemolytic zoonotic pathogen that can lead to fatal medical conditions. The most common routes of transmission are attributed to animal contact and to the consumption of unpasteurized dairy products. The current treatment advice is a combination of medical and surgical intervention. 1
